# Ex-ante online risk assessment for building emergency evacuation through multimedia data

**DOI:** 10.1371/journal.pone.0215149

**Published:** 2019-04-11

**Authors:** Haoran Zhang, Xuan Song, Xiaoya Song, Dou Huang, Ning Xu, Ryosuke Shibasaki, Yongtu Liang

**Affiliations:** 1 Center for Spatial Information Science, The University of Tokyo, Kashiwa-shi, Chiba, Japan; 2 Heilongjiang Cold Region Urban-Rural Human Settlements Science Key Laboratory, Harbin Institute of Technology, Nangang District, Harbin, Heilongjiang Province, China; 3 Beijing Key Laboratory of Urban oil and Gas Distribution Technology, China University of Petroleum-Beijing, Changping District, Beijing, China; The University of Warwick, UNITED KINGDOM

## Abstract

Ex-ante online risk assessment for building emergency evacuation is essential to protect human life and property. Current risk assessment methods are limited by the tradeoff between accuracy and efficiency. In this paper, we propose an online method that overcomes this tradeoff based on multimedia data (e.g. videos data from surveillance cameras) and deep learning. The method consists of two parts. The first estimates the evacuee position as input for training the assessment model to then perform risk assessment in real scenarios. The second considers a social force model based on the evacuation simulation for the output of training model. We verify the proposed method in simulation and real scenarios. Model sensitivity analyses and large-scale tests demonstrate the usability and superiority of the proposed method. By the method, the computation time of risk assessment could be decreased from 10 minutes (by traditional simulation method) to 2.18 s.

## Introduction

Building emergency evacuation is an indispensable process to protect human life and property under the occurrence of events such as fires, earthquakes, and terrorist attacks [1,2]. Statistics show that emergencies causing numerous casualties mostly occur by either the lack of reliable evacuation facilities or mismanagement for safe and timely evacuation, especially in crowded public places such as shopping malls, stadiums, theatres, and other entertainment venues. Employing more evacuation guiders and facilities seems the direct way to avoid the casualties but also clumsy and high-cost. If security administrators can be informed about the real-time potential risk, they can take the relatively suitable ex-ante measures (adding more guiders and facilities) and avoid the overreaction or the negligence hence to save the cost and improve safety. Therefore, ex-ante online risk assessment for building emergency evacuation can be greatly useful to guide decision-making for extreme event prevention, mitigation, preparedness, and response [3,4].

Nevertheless, ex-ante online risk assessment is a highly complex problem [5]. For instance, the risk assessment system architecture for building emergency evacuation is generally based on specific criteria of safety design. Depending on the number of exits, stairways, and the structural layout of a building, safety measures determine the ability for a smooth and timely evacuation. However, this kind of method does not consider spatiotemporal uncertainties of the initial evacuee position. In addition, emergency evacuation does not only depend on the building structure but is also affected by the number and initial position of evacuees within the building. Therefore, the corresponding multimedia data mining to assess risk are complex yet essential aspects for successful emergency evacuation.

Another evacuation problem is the demand to respond. The crowded state in a building represents a rapidly varying process, and emergencies occur randomly and instantaneously. Thus, an ex-ante online risk assessment system is necessary to continuously determine the safety state of a building based on, for instance, varying visual information. Although some studies showed that large-scale data training could accelerate risk assessment for complex systems [6], generally, we do not have enough data from real emergency events to tune the corresponding algorithms.

**Fig 1:**
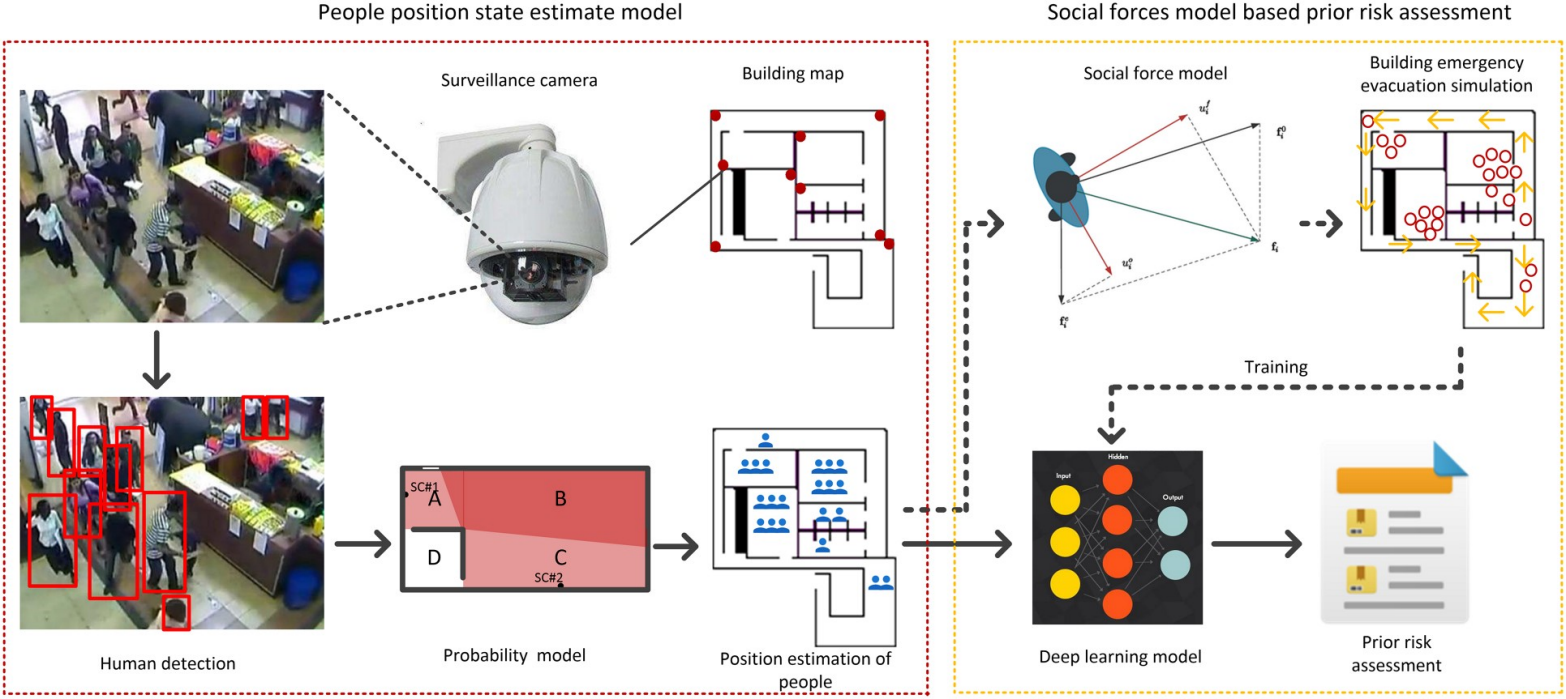
Diagram of proposed real-time risk assessment method for building emergency evacuation.

In this paper, we propose an ex-ante online risk assessment method for building emergency evacuation, as illustrated in Fig 1. The proposed method exhibits a general computational framework and techniques for solving risk metrics with a certain complexity. In addition, the method consists of two main parts, (1) a method to estimate the initial evacuee position as input for training the deep learning assessment model; (2) a social force model for evacuation simulation as output for training. Specifically, according to historical videos retrieved from surveillance cameras in buildings, the online occupation in each area can be estimated using a human detection algorithm and a proposed possibility method. Subsequently, the social force model allows to predict the emergency evacuation process and evaluate potential risks. With thousands of times of simulation, we obtain a large-scale dataset, which is used to train a deep learning model and determine the online model for risk assessment and evacuation. Compared with previous studies, the proposed method provides evacuation prediction strictly based on real-time information, which ensures the reliability of the input parameters for risk assessment. In addition, multi-scenario sensitivity analysis based on the structural layout of a building and dynamic properties can be implemented using the proposed method. This feature can provide guidelines based on data mining to support future research on interior design and infrastructure optimization. Moreover, the proposed method considers the uncertainty caused by blind spots of the camera system, thus making it more practical and realistic than previous similar developments.

The remainder of this paper is organized as follows. In Section II, we present an overview of related works. The estimation model to determine the number and position of evacuees is detailed in Section III. In Section IV, we describe the risk assessment model. Then, an evaluation procedure for different scenarios is presented in Section V. Section VI reports experiments and results in a real scenario. Finally, we draw conclusions and provide future research directions in Section VII.

## Related works

The current demand for smart and safe city development makes emergency evacuation one of the hottest topics in fields including risk management, public health, and urban planning. In fact, over 22 thousand articles report emerging applications, discussions, and methodologies related to this topic within the last 5 years[7,8]. Most studies have focused on probabilistic risk models, simulations [9], evacuation drills [10], and socio-psychological aspects of documented disasters [11]. However, the design of online risk assessment systems has been scarcely addressed. The existing approaches can be divided into three types, namely, analyses based on building the structure, evacuees distribution, and data.

In a large-space building, the connectivity among indoor areas is crucial to determine emergency evacuation, because it reflects the building accessibility. Existing methods based on building structure primarily focus on static spatial information, and disregard dynamic information. Furthermore, such methods usually neglect the multi-story aspects that include elements such as stairways, which are essential for successful evacuation.

To overcome these limitations, several studies have been focused on developing simulation models that consider evacuees’ motion. These methods can be classified as models using 1) logical rules [12], 2) statistics [13], 3) analytical prediction [14], and 4) agent interactions [15]. Lv, Huang [16] propose an integer programming (ILP) method for emergency evacuation management. Likewise, Zhang, Liu [17] design a mixed-integer programming model and a heuristic algorithm using network optimization and diffusion simulation for emergency evacuation. Ha and Lykotrafitis [18] proposed a social force model to investigate the effect of complex building structures during an emergency evacuation. However, these simulation-based methods demand a long computation time, thus being unsuitable for online risk assessment. Moreover, these studies usually propose predictions with a high dependency on detailed and accurate input parameters and disregard their application to real scenarios. Focusing on the merge flow at the stairs during building emergency evacuation, Wu and Huang [19] proposed a control volume model for modelling the dynamics of the evacuees and derive the evacuation times. However, these simulation-based methods demand a long computation time, thus being unsuitable for online risk assessment. Moreover, these studies usually propose predictions with a high dependency on detailed and accurate input parameters and disregard their application to real scenarios.

In contrast, data-driven approaches can reflect more realistic situations and comprise a promising research direction on emergency evacuation[20]. For instance, Liu and Jabari [21] propose a data-driven method for online traffic management under emergency evacuation. They developed evacuation software with an embedded geographic information system allowing users to build evacuation scenarios and test heuristic algorithms for evacuation. Yuan, Liu [22] present a dynamic data-driven approach to describe driving variability under both normal and emergency scenarios. However, these data-driven methods are mainly aimed for large-scale evacuation processes, and building evacuation as the elementary unit for mass emergency evacuation is seldom considered.

Overall, previous studies were limited by the tradeoff between usability and theoretical accuracy. Although novel data-driven methods have been developed to effectively handle this tradeoff, more research on the strength of higher-quality sample sets and the underlying solutions for evacuation is still required.

## Estimation of evacuees status

The model to estimate evacuee status aims to determine the number and position of evacuees within a building. Although surveillance videos can provide plenty of visual information, additional intensive processing is required. There are several image-based human detection methods currently available, such as Faster R-CNN [23] and Mask R-CNN [24], which are able to quickly determine the number of people by using neural networks. In fact, object detection is a complex and important field of computer vision and pattern recognition [25–27]. Therefore, we limit ourselves to employ the available human detection method in the proposed method for risk assessment and do not discuss this aspect in detail.

**Fig 2:**
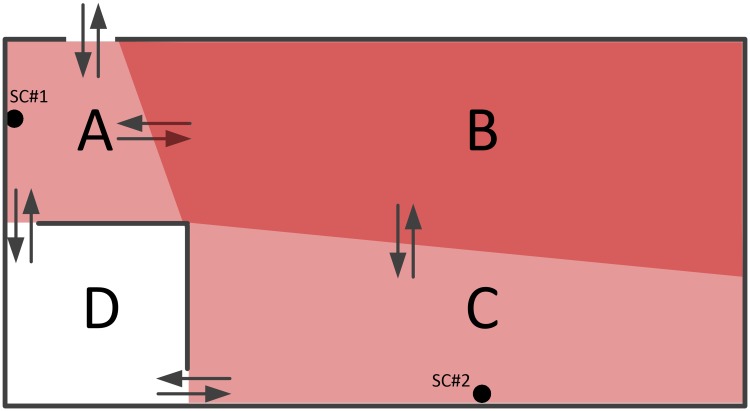
Coverage of surveillance cameras SC#1 and SC#2.

Although the number of people within a building can be estimated from surveillance videos using any consolidated method, the position estimation of each evacuee within the building is a challenging problem. In fact, surveillance cameras usually fail to cover every area of the building, and hence several blind spots arise, thus impeding to obtain complete visual information of people in those areas. When implementing emergency evacuation measures, this incomplete information may undermine the process and risk assessment. On the other hand, surveillance cameras usually provide overlapping coverage. Therefore, this redundant visual information affects the estimation of people status in the building. Fig 2 illustrates a scenario where two surveillance cameras, SC#1 and SC#2, have overlapping coverage. Camera SC#1 covers areas A and B, and SC#2 covers areas B and C. Hence, area B has overlapping coverage, whereas area D, which is enclosed by walls, is a blind spot. From the surveillance videos of cameras SC#1 and SC#2, we can estimate the number of people in areas A + B and C + B, respectively, but the number of people per area is unknown.

Therefore, we developed an estimation model considering overlapping coverage by using the temporal information from the surveillance videos to overcome the spatial redundancy. Specifically, we track people over discrete time instants and use a conservation law. Hence, the next-instant number of people in an area is equal to the number in the current instant plus the total number of entering people minus the total number of leaving people:

$$N_{\text{S}}{}_{a,b+1}=N_{\text{S}}{}_{a,b}+\sum_{a^{\prime }}\left(N_{\text{C}}{}_{a^{\prime },a,b}-N_{\text{C}}{}_{a,a^{\prime },b}\right),$$ (1)

∀*a*, *a*′∈ *A* and ∀*b* ∈ *B*, where *A* is the set of individual areas, *B* is the set of time instants, *N*_S*a*,*b*_ is the estimated number of people at area *a* and time *b*, and *N*_C*a*,*a*′,*b*_ is the number of people that went from area *a* to *a*′ at the time *b*.

We assume that the number of people going among different areas satisfies a normal distribution with the mean being the number of people passing through an area multiplied by a transference probability, whose value and that of the variance can be obtained from indoor investigation and statistics.

$$N_{\text{C}}{}_{a,a^{\prime },b}∼N\left(\alpha_{a,a^{\prime }}N_{\text{S}}{}_{a,b},\sigma_{a,a^{\prime },b}{}^{2}\right),$$ (2)

where *α*_*a*,*a*′_ is the transference probability from area *a* to *a*′ and *σ*_*a*,*a*′,*b*_ is the transference variance.

By considering the distribution in (2) into (1), the difference between the number of people in area *S*_i_ among two consecutive instants satisfies normal distribution

$$N_{\text{S}}{}_{a,b+1}-N_{\text{S}}{}_{a,b}∼N\left(\mu_{a,b},\eta_{a,b}{}^{2}\right)$$ (3)

where

$$\mu_{a,b}=\sum_{a^{\prime }}\left(\alpha_{a^{\prime },a}N_{\text{S}}{}_{a^{\prime },b}-\alpha_{a,a^{\prime }}N_{\text{S}}{}_{a,b}\right),$$ (4)

$$\eta_{a,b}=\sum_{a^{\prime }}\left(\sigma_{a^{\prime },a,b}-\sigma_{a,a^{\prime },b}\right).$$ (5)

Therefore, we can calculate the transference probability from the estimated difference in the number of people among consecutive instants as follows:

$$P_{a,b}=\frac{1}{\sqrt{2\pi }\eta_{a,b}}\text{exp}\left\{-\frac{{\left[\left({\tilde{N}}_{\text{S}a,b+1}-{\tilde{N}}_{\text{S}a,b}\right)-\mu_{a,b}\right]}^{2}}{2\eta_{a,b}{}^{2}}\right\},$$ (6)

where ${\tilde{N}}_{\text{S}i,t}$ is the real number of people at area *i* and time *t*.

Finally, we consider the total probabilities of all individual areas as an objective function for maximization, and the conservation law as constrain. The resulting nonlinear programing model is expressed by (7), which can be solved by evolutionary algorithms such as particle swarm optimization.

$$\begin{array}{c} \begin{array}{c} {\tilde{N}}_{\text{S}}=\text{argmax(}\sum_{a}P_{a}\text{)}\\ \text{s}.\text{t}.\sum_{a*}{\tilde{N}}_{\text{S}a*}=N_{\text{C}}{}_{k} \end{array} \end{array},$$ (7)

∀a* ∈ *A*_C*k*_ and ∀*k* ∈ *K*, where *A*_C*k*_ is the set of individual areas covered by a surveillance camera *k*, *K* is the set of surveillance cameras, and *N*_C*k*_ is the number of people estimated from the video of a surveillance camera *k*.

## Risk assessment

The proposed risk assessment method is composed of a social force model to compute the output of a training model, which maps the evacuees’ state to limit the time for evacuation.

### Social force model

We propose an agent-based model considering social forces to represent building emergency evacuation, where the agents are the people within the building. Based on historical surveillance videos and the estimation model proposed in Section II, we randomly generated a certain number of agents distributed over the areas of a building to determine a case study. Each agent is influenced by its dynamic surroundings, including walls, obstacles, and other agents. The complexity of these interactions usually demands mathematical abstractions to obtain a suitable representation. Three kinds of forces act between agents and their environment [28]: 1) desired direction force *f*_D*i*_, 2) repulsive force *f*_R*i*,*i*′_ and 3) wall force *f*_W*i*,*w*_, which contributes to the variation of agent velocity over time:

$$m_{i}\frac{\text{d}v_{i}}{\text{d}t}=f_{\text{D}i}+\sum_{i^{\prime }(\neq i)}f_{\text{R}i,i^{\prime }}+\sum_{w}f_{\text{W}i,w},$$ (8)

∀*i*,*i*′ ∈ *I* and ∀*w* ∈ *W*, where *I* is the set of agents, *W* is the set of walls and obstacles, *m*_*i*_ is the mass of agent *i*, and *v*_*i*_ is its moving speed.

Furthermore, an agent has a desired direction *e*_i_* along which it prefers to walk with desired speed *v*_i_*. Therefore, the desired direction force is given by

$$f_{\text{D}i}=m_{i}\frac{v_{i}*\left(t\right)e_{i}*\left(t\right)-v_{i}\left(t\right)}{\tau },$$ (9)

where *τ* is the reaction time.

For psychological and cultural reasons, people prefer to keep some distance to others. Therefore, the relative position among agents also impacts their trajectory, which can be described by repulsive force

$$\begin{array}{ll} f_{\text{R}i,i^{\prime }} & =\left\{\alpha_{i}\text{exp}\left[\left(r_{i,i^{\prime }}-d_{i,i^{\prime }}\right)/\beta_{i}\right]+kg\right\}n_{i,i^{\prime }}\\ & +\kappa g\Delta v_{i^{\prime },i}^{t}t_{i,i^{\prime }} \end{array},$$ (10)

$$g=\{\begin{array}{ll} 0 & \text{if}\left(r_{i,i^{\prime }}<d_{i,i^{\prime }}\right)\\ r_{i,i^{\prime }}-d_{i,i^{\prime }} & \text{else} \end{array},$$ (11)

where *r*_*i*,*i*′_ = *r*_*i*_ + *r*_*i′*_ is the sum of radii considered for agents *i* and *i*′, *d*_*i*,*i*′_ = ||*l*_*i*_ −*l*_*i*′_|| is the distance between agents *i* and *i*′, $n_{i,i^{\prime }}=\left(n_{i,i^{\prime }}^{1},n_{i,i^{\prime }}^{2}\right)=\left(l_{i}-l_{i^{\prime }}\right)/d_{i,i^{\prime }}$ is the normalized vector between agents, $t_{i,i^{\prime }}=\left(-n_{i,i^{\prime }}^{2},n_{i,i^{\prime }}^{1}\right)$ is the tangential direction, $\Delta v_{i^{\prime },i}^{t}=\left(v_{i^{\prime }}-v_{i}\right)t_{i,i^{\prime }}$ is the tangential velocity and the other variables represent model parameters.

Analogous to the repulsive force, the wall force can be expressed as [29]

$$f_{\text{W}i,w}=\left\{\alpha_{i}\text{exp}\left[\left(l_{i}-d_{i,w}\right)/\beta_{i}\right]+kg\right\}n_{i,w}+\kappa g\Delta v_{i}t_{i,w},$$ (12)

$$g=\{\begin{array}{ll} 0 & \text{if}\left(l_{i}\leq d_{i,w}\right)\\ l_{i}-d_{i,w} & \text{else} \end{array}.$$ (13)

### Training model

Here, we use deep learning model to determine the relation between the initial agent distribution and risk assessment for building emergency evacuation. The network input is the number of people detected by the video of surveillance cameras. Generally, they will have a formulation that can map input ***X***, all the way to the target objective, ***Y***, via a series of hierarchically stacked operations. Those operations are typically linear operations *W*_*i*_, followed by a non-linearities *f*_*i*_, like so:

$$Y=f_{N}\left(\cdots f_{2}\left(f_{1}\left(X^{T}W_{1}\right)W_{2}\right)\cdots W_{3}\right)$$ (14)

Optimization algorithms play an important role in deep learning. They help us to find a proper set of parameters for our model. The problem of minimizing an objective function is generally expressed as:

$$L\left(\theta \right)=\frac{1}{n}\sum_{i=1}^{n}L_{i}\left(\theta \right)$$ (15)

where *θ* is the parameter, including *W* and *B* at each layer, need to estimate which can minimize *L*(*θ*). *n* is the size of data sthe et used for training.

Stochastic gradient descent (SGD) is a simple yet very efficient approach for minimizing an objective function by iterating gradient descent. Even though SGD has been proposed for a long time, it is still widely used and effective approach in machine learning. In SGD, the gradient iteration is done as:

$$\theta_{t+1}=\theta_{t}-\alpha \nabla L\left(\theta_{t}\right)$$ (16)

where *α* is the learning rate. By iteration along the gradient, SGD will converge into a stable, or minimization status.

Adagrad is an algorithm for gradient-based optimization which adapts the learning rate to the parameters, performing larger infrequent and smaller updates for frequent parameters.

$$\theta_{t+1}=\theta_{t}-\frac{\alpha }{\sqrt{G_{t}+\varepsilon }}\nabla L\left(\theta_{t}\right)$$ (17)

where *G*_*t*_ is a diagonal matrix and *ϵ* is a smoothing term.

RMSprop devides the learning rate by an exponentially decaying average of squared gradients.

$$g_{t}=\nabla L\left(\theta_{t}\right)$$ (18)

$$E{\left[g^{2}\right]}_{t}=0.9E{\left[g^{2}\right]}_{t-1}+0.1g_{t}^{2}$$ (19)

$$\theta_{t+1}=\theta_{t}-\frac{\alpha }{\sqrt{E{\left[g^{2}\right]}_{t}+\varepsilon }}g_{t}$$ (20)

Adaptive Moment Estimation (Adam) is another method that computes adaptive learning rates for each parameter.

$$m_{t}=\beta_{1}m_{t-1}+\left(1-\beta_{1}\right)g_{t}$$ (21)

$$v_{t}=\beta_{2}v_{t-1}+\left(1-\beta_{2}\right)g_{t}^{2}$$ (22)

$$\theta_{t+1}=\theta_{t}-\frac{\alpha }{\sqrt{\frac{v_{t}}{1-\beta_{2}^{t}}}}\frac{m_{t}}{1-\beta_{1}^{t}}$$ (23)

Adamax scales the *v*_*t*_ factor in the Adam update to the *l*_*p*_ norm.

## Method evaluation

**Fig 3:**
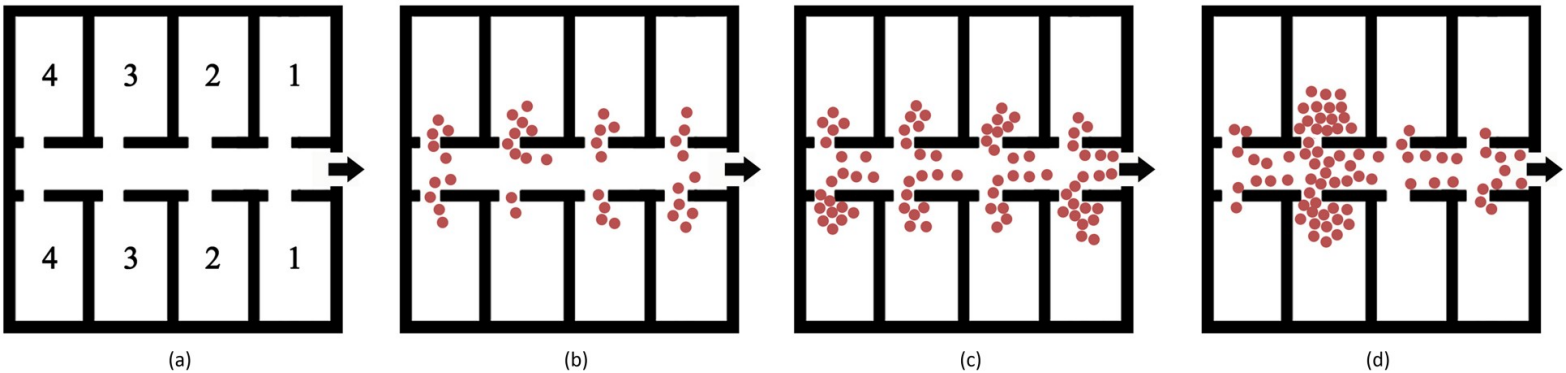
(a) Simulated evacuation scenario in an office building. Evacuation for (b) *N* = 40, *MD* = 14.00; (c) *N* = 80, *MD* = 14.00; (d) *N* = 80, *MD* = 15.25. (*MD*: mean distance to the exit).

To evaluate the proposed method considering its sensitivity with respect to both the number of evacuees and their positions during building emergency evacuation, we implemented a simulation scenario that consists of a single-floor building with eight offices and one exit, as shown in Fig 3(a). Sorting by the mean distance between the centre of each room and exit, four kinds of room are numbered.

### Sensitivity to number of evacuees

**Fig 4:**
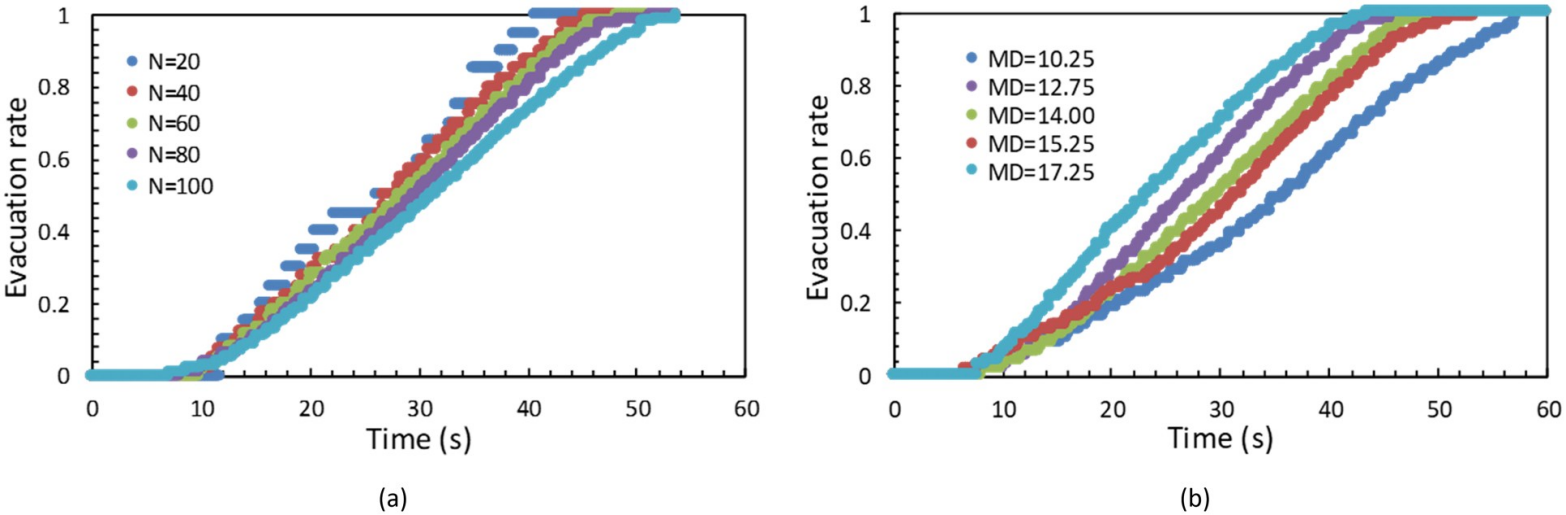
Sensitivity according to (a) number of evacuees and (b) evacuee positions.

We first evaluated the influence of the number of evacuees on the evacuation process and risk assessment. We verified a number of evacuees, *N*, of 20, 40, 60, 80, and 100. Fig 3(b) and 3(c) illustrate the evacuation state of 40 and 80 people at 10 s after emergency happening (red dots represent agents). The evacuation rate is shown in Fig 4(a). The last person leaves the building at 40.64 s (*N* = 20), 45.20 s (*N* = 40), 48.24 s (*N* = 60), 51.2 s (*N* = 80) and 53.68 s (*N* = 100). When there are less than 40 evacuees, the evacuation rate rapidly increases, indicating no serious congestion during the evacuation. The evacuation for 40, 60, and 80 people show a similar trend, whereas, for 100 evacuees, the rate increases slower, thus indicating serious congestion.

### Sensitivity to evacuee positions

The initial evacuee positions might also influence the evacuation process and risk assessment. Hence, we considered the mean distance to the exit, *MD*, to represent the evacuee positions, and tested simulation scenarios with *MD* of 10.25 m, 12.75 m, 14.00 m, 15.25 m, and 17.25 m. The evacuation state for *MD* of 14.00 m and 15.25 m at 10 s are illustrated in Fig 3(c) and 3(d), respectively. The evacuation rate according to the distance to the exit *MD* is shown in Fig 4(b). The last person leaves the building at 43.44 s (*MD* = 10.25 m), 46.20 s (*MD* = 12.75 m), 51.20 s (*MD* = 14.00 m), 53.28 s (*MD* = 15.25 m) and 57.20 s (*MD* = 17.25 m). In fact, a large *MD* implies an unbalanced initial distribution of evacuees and serious congestions. Furthermore, risk measure *RC* exponentially grows with *MD*, and the sensitivity to *MD* is higher than that to *N*.

The abovementioned sensitivity analyses also highlight the importance of the initial position of evacuees, their number, and the dynamic indoor environment for risk assessment of potential building emergency evacuation.

## Real-scenario results

### Experiment scenario

**Fig 5:**
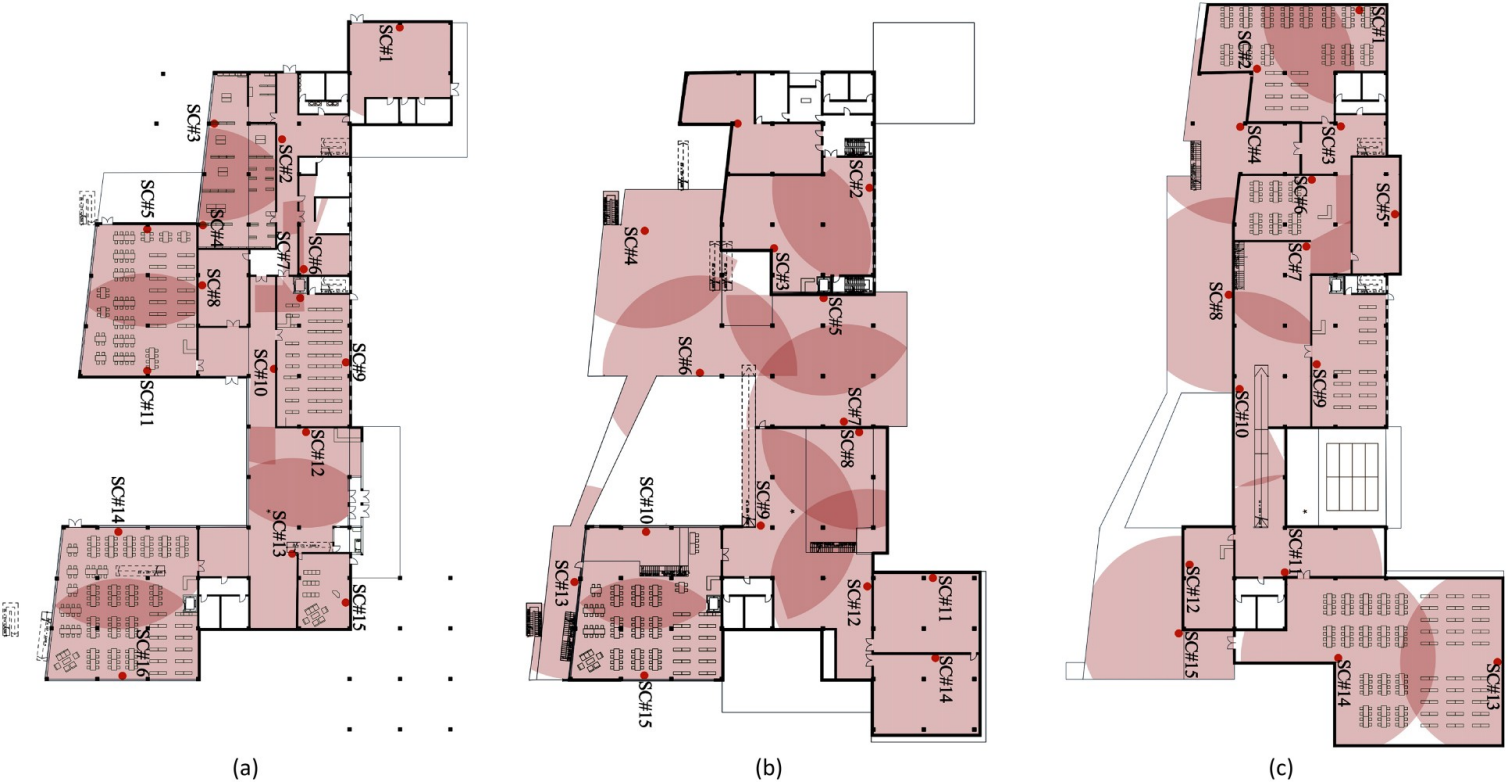
Structure of a library used to evaluate the proposed method: (a) first, (b) second, and (c) third floors.

We evaluated the proposed method on a real scenario, namely, a three-floor library in Taiyuan, China, whose structure, surveillance camera locations and coverage are shown in Fig 5. This building is designed based on the criterion (Code for Fire Protection Design of Buildings, GB50016). The limit time of evacuation is 2 minutes. The building contains 46 surveillance cameras (1^st^ floor: 16; 2^nd^ floor: 15; 3^rd^ floor: 15), which can rotate 360° and have a working radius of approximately 15 m. The coverage of the cameras (transparent red areas) show overlapping coverage (darker red areas) and blind spots (white areas). There are nine exits at the 1^st^ and two on the 2^nd^ floor, where an outdoor platform is available. In addition, four stairways connect the 1^st^ and 2^nd^ floors, and six connect the 2^nd^ and 3^rd^ floors. Bold black lines represent solid walls, and thin black lines are for edges of the cameras (transparent red areas) show overlapping coverage (darker red areas) and blind spots (white areas). Due to the lack of complementary data and additional video material on escape panics, the evacuation process generated by the social force model cannot be validated. Our model is established based on the trust of the accuracy of the social force model which has been widely validated by multiple experiments[30].

### Evacuation simulation

**Fig 6:**
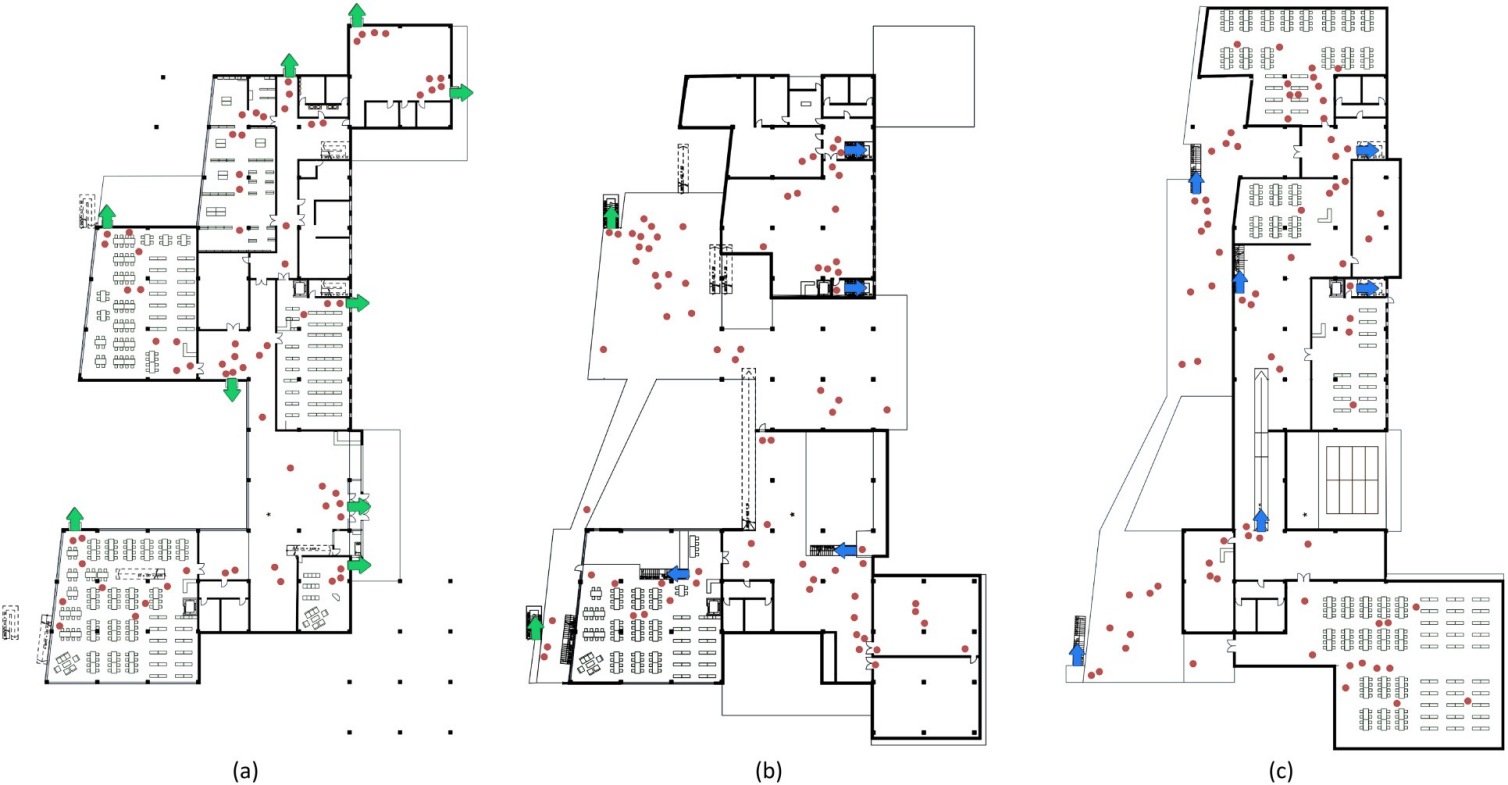
Evacuation process at the (a) first, (b) second, and (c) third floors.

**Fig 7:**
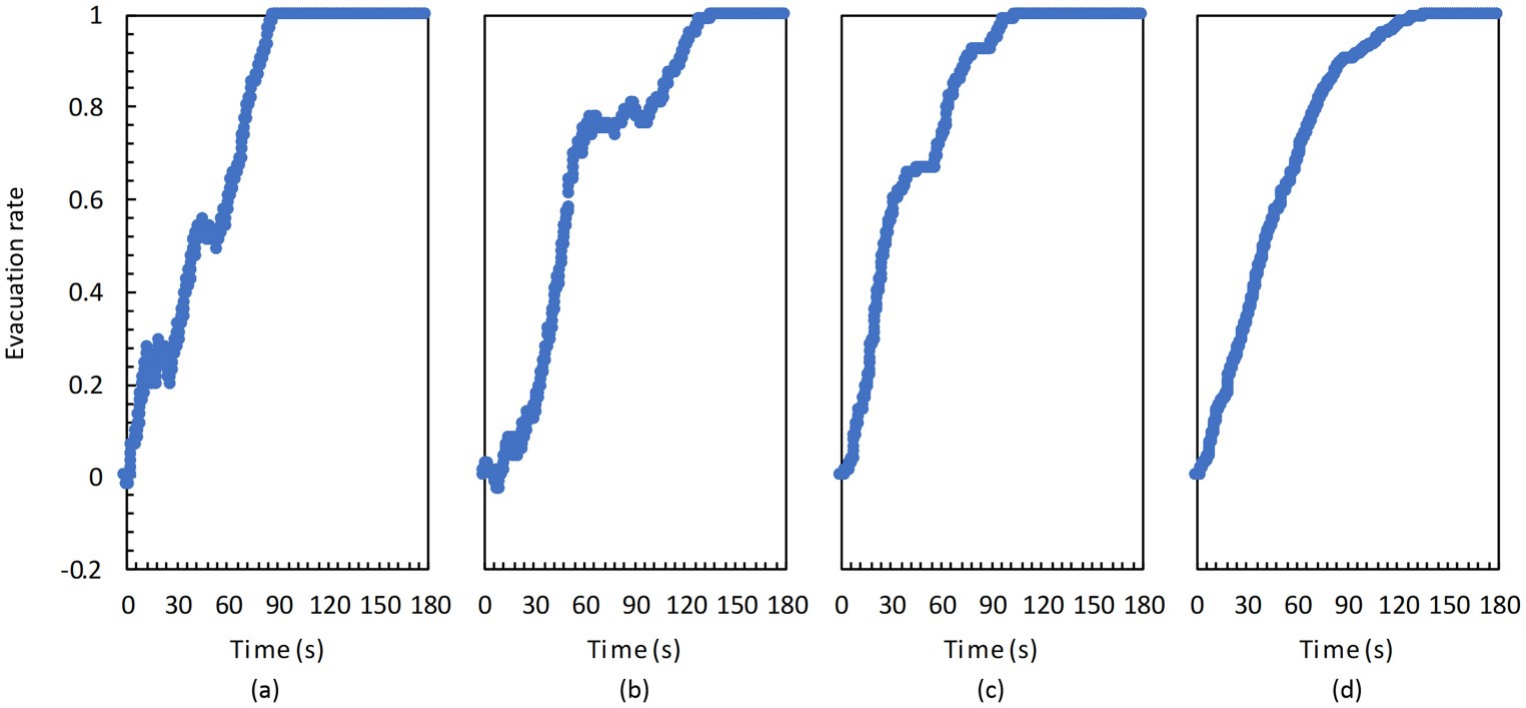
Varying of evacuation rate in the real scenario of (a) first, (b) second, and (c) third floors as well as (d) whole building.

We considered a situation retrieved from real video footage as an example. Specifically, we calculated the initial position of people by using historical surveillance videos, the Faster R-CNN object detection method, and the probability model proposed in Section III. A resulting emergency evacuation process aimed to last 10 s is illustrated in Fig 6 (red dots represent agents). The green arrows represent the exits, and the blue ones represent descending stairways. We can see that every agent tries to find the shortest evacuation path to leave the building. The varying of evacuation rate in this example is shown in Fig 7. There are some decreases occurring in the evacuation rates of the first and second floors, and even they get negative at the beginning of evacuation. The reason of that is, during the evacuation, the people in the higher floors will run to downstairs, and if the entering number is more than the leaving number for a floor, the evacuation rate would be decreased at this moment. As there are two exits at the 2^nd^ floor and those are the nearest for the 3^rd^ floors people, the evacuation rate of the 2nd floor is the last to get to 1. At 136.48 s, all of the people have evacuated out from the building.

### Training results

In our experiment, we build a neural network which contains an input layer, *NL* = 3, 4, 5, 6 hidden layers and an output layer. The input data is set to be the distribution of the number of pethe rson in grids. Meanwhile t,he output data is set to be the number of pethe rson who escape from the building successfully in timestamps.

**Fig 8:**
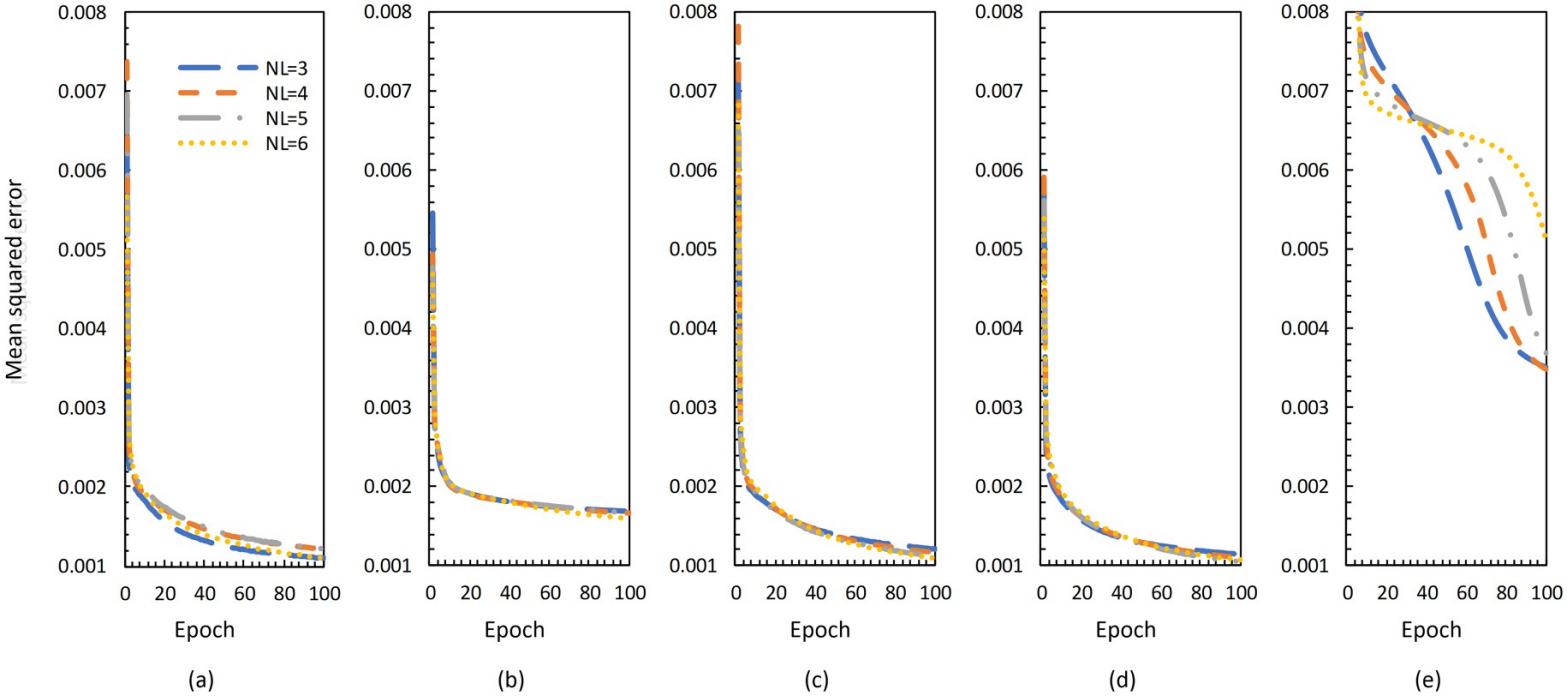
Convergence process of (a) Adam, (b) Adagrad (c) Adamax (d) RMSprop and (e) sgd.

We use 10,000 normalized training samples to train our model, and the loss function is set to be the classical mean squared errors. From Fig 8, we can see the model reach a fast convergence except for the SGD optimizer.

Then we evaluate the accuracy of our model using another 5,000 samples, as shown in Table 1. In our experiment, we regard the predicted result as correct if the difference between the result of prediction and the true value is less than 10% of true value. Then we get the best accuracy of our model is about 95.78%, with 3 hidden layers using Adamax optimizer. The computation time of once social force model simulation is about 10 minutes, while, solving by the proposed method, the computation time is just 2.18 s.

**Table 1:** Accuracy of different methods.

NL	3	4	5	6
**Adam**	95.34%	95.32%	95.22%	94.94%
**Adagrad**	95.24%	95.74%	95.58%	95.68%
**Adamax**	95.78%	95.04%	95.46%	95.74%
**RMSprop**	94.58%	94.38%	94.64%	94.82%
**sgd**	72.82%	73.16%	68.56%	47.12%

**Fig 9:**
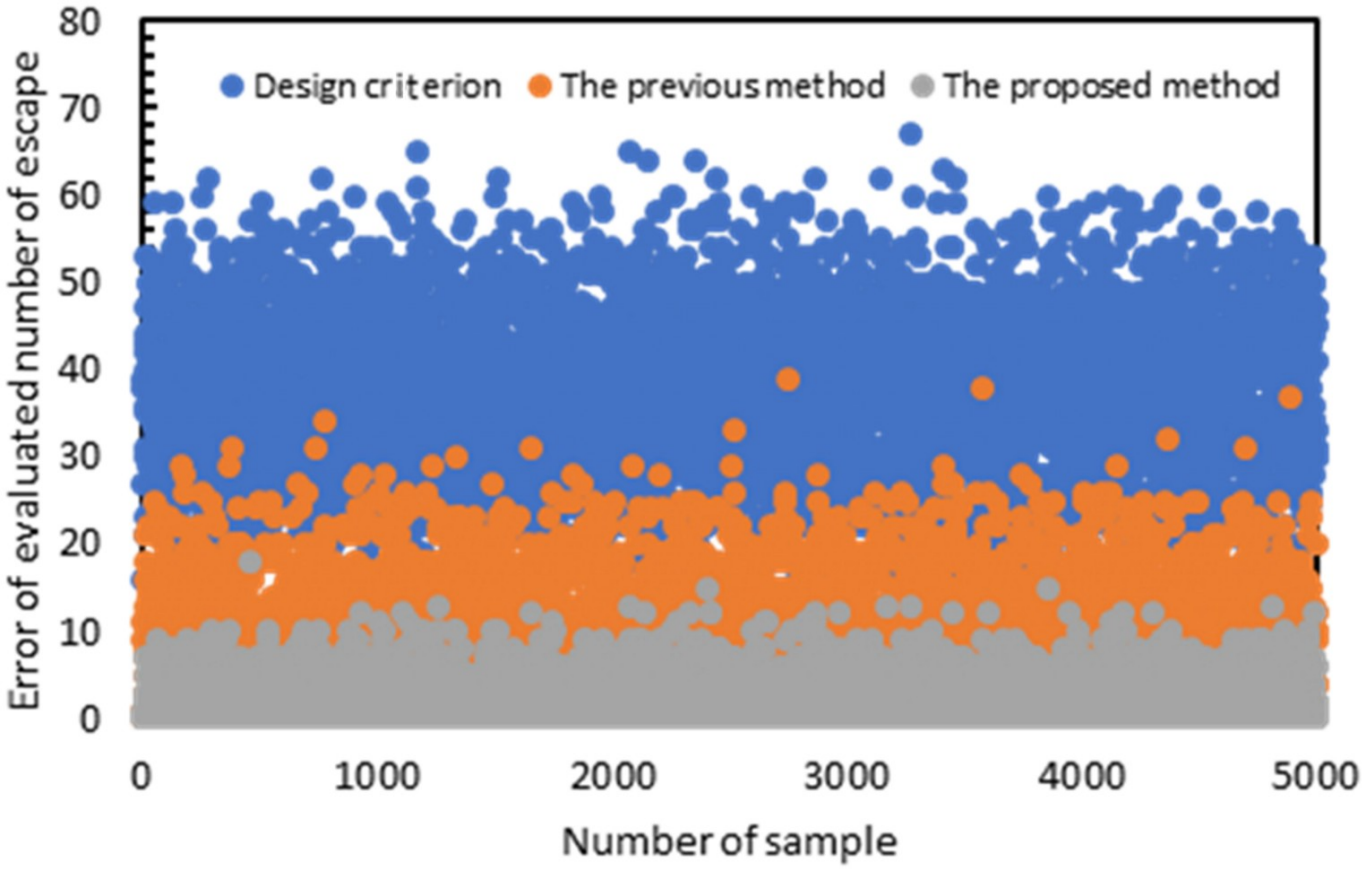
Error of evaluated number of escape.

In addition, we made a comparison analysis with the evaluated number of escape by design criterion (number of people who have not left the building at 2 minutes) and previous method [19] using another 5,000 samples, as shown in Fig 9. From, the results, we can find that the accuracy of the proposed method is much higher than others. The design criterion does not take the uncertainties of the evacuation process and congestion into consideration, therefore, there is a large error in the evaluated number of escape. To the previous method [19], although it has considered dynamic evacuation process at stairs, it does not take the evacuation process from the initial position to the stairs into the simulation model. Therefore, the errors of this method are also larger than the proposed method.

## Conclusions

In this paper, we propose an ex-ante online risk assessment method for building emergency evacuation. The general framework of the proposed method is detailed, and it allows to provide fast response and blind spot detection. By analyzing the model sensitivity and its performance in a real scenario, we demonstrate the usability and performance of the proposed method. In addition, the results from the real scenario show that the proposed method can perform risk assessment in complex settings.

There are certain limitations in this method. The method is limited by the complexity of the process and time-consuming training. Moreover, since the method is not a generalized model which means the model needs to be retrained when adopted in a new building environment, to the emergency management engineer, the model is not easily exercisable. Additionally, although the accuracy of the social force model in the escape panic condition has been validated, some data-driven methods such as the Long Short Term Memory (LSTM) based pedestrian dynamics model, shows more advantages in simulating the interactions among the pedestrians which can detailly reveal the evacuation process.

In future works, we will pursue the following topics to improve our system. (1) Introducing the transfer learning method to increase the training speed and make the model easier to be adopted in a new building environment. (2) Developing the data-driven method to promote the accuracy of the pedestrian dynamics simulation. (3) Calling for complementary data and additional video material on escape panics to test our model quantitatively and compare it with alternative models.
